# Understanding Public Perceptions of Virtual Reality Psychological Therapy Using the Attitudes Towards Virtual Reality Therapy (AVRT) Scale: Mixed Methods Development Study

**DOI:** 10.2196/48537

**Published:** 2024-01-12

**Authors:** Aislinn D Gomez Bergin, Aoife M Allison, Cassie M Hazell

**Affiliations:** 1 National Institute of Health and Care Research MindTech MedTech Co-operative Institute of Mental Health University of Nottingham Nottingham United Kingdom; 2 National Institute of Health and Care Research Nottingham Biomedical Research Centre University of Nottingham Nottingham United Kingdom; 3 Mental Health and Clinical Neurosciences School of Medicine University of Nottingham Nottingham United Kingdom; 4 School of Computer Science University of Nottingham Nottingham United Kingdom; 5 Department of Psychological Interventions School of Psychology University of Surrey Guilford United Kingdom

**Keywords:** psychological interventions, digital, virtual reality, virtual agent, mental health, presence

## Abstract

**Background:**

Virtual reality (VR) psychological therapy has the potential to increase access to evidence-based mental health interventions by automating their delivery while maintaining outcomes. However, it is unclear whether these more automated therapies are acceptable to potential users of mental health services.

**Objective:**

The main aim of this study was to develop a new, validated questionnaire to measure public perceptions of VR therapy (VRT) guided by a virtual coach. We also aimed to explore these perceptions in depth and test how aspects such as familiarity with VR and mental health are associated with these perceptions, using both quantitative and qualitative approaches.

**Methods:**

We used a cross-sectional mixed methods design and conducted an exploratory factor analysis of a questionnaire that we developed, the Attitudes Towards Virtual Reality Therapy (AVRT) Scale, and a qualitative content analysis of the data collected through free-text responses during completion of the questionnaire.

**Results:**

We received 295 responses and identified 4 factors within the AVRT Scale, including attitudes toward VRT, expectation of presence, preference for VRT, and cost-effectiveness. We found that being more familiar with VR was correlated with more positive attitudes toward VRT (factor 1), a higher expectation of presence (factor 2), a preference for VRT over face-to-face therapy (factor 3), and a belief that VRT is cost-effective (factor 4). Qualitative data supported the factors we identified and indicated that VRT is acceptable when delivered at home and guided by a virtual coach.

**Conclusions:**

This study is the first to validate a scale to explore attitudes toward VRT guided by a virtual coach. Our findings indicate that people are willing to try VRT, particularly because it offers increased access and choice, and that as VR becomes ubiquitous, they will also have positive attitudes toward VRT. Future research should further validate the AVRT Scale.

## Introduction

### Background

Virtual reality (VR) is an immersive environment where people can interact using either computer equipment, such as a screen and mouse, or VR-enabled headsets and controllers, where additional sensors can track the users’ actions in real time. The latter application provides people with a greater sense of presence, a term used to describe how closely a virtual environment is interpreted as real [1]. In recent years, VR has been used successfully in a range of health care settings to improve and increase access to treatment [2]. In particular, VR has been used in the delivery of psychological therapies for a range of mental health problems, with several decades of evidence demonstrating its clinical efficacy in the treatment of psychosis, depression, anxiety, and eating disorders [3-8].

VR therapies (VRTs) were initially developed to be used by therapists as an adjunct or tool in their delivery of therapy. However, the need for a real-world therapist to deliver VRTs presents a key challenge for their widespread implementation [9]. Researchers have shown that the automation of some therapeutic elements may overcome this barrier to meet the increase in demand for treatment globally [10,11]. Emerging evidence demonstrates that VRTs can be successfully delivered with little to no therapist involvement, with virtual coaches supporting people receiving therapy for fear of heights and agoraphobia in the context of psychosis [10,11]. Virtual coaches are also known as virtual agents [12]. These characters are not under human control and therefore offer automation of therapies, in which dialogue and responses are scripted instead of the formulation that is offered by real-world therapists.

There are financial and resource incentives for mental health services to offer more automated therapies [13]. Clinicians also appear to be in support of VRTs. For example, cognitive behavior therapists [14] and psychiatric health care staff [15] reported positive attitudes toward VRT, particularly when they were more familiar with VR. However, these studies do not consider how staff feel about VRT guided by a virtual coach and, notably, do not explore patient and public perceptions of VRT. VRT dropout rates have been used as a proxy measure of patient experience, and these figures show similar dropout rates to therapies delivered without VR [16]. However, dropout rates from research does not provide us with a clear picture of whether people will engage in therapies delivered using VR, including those guided by a virtual coach. A content analysis of social media posts by the public appears to suggest an interest in the application of VR in mental health care [17]. Staff and service users also have positive views toward their use in mental health inpatient facilities [18]. However, these studies still do not directly ask potential users of mental health services whether they would be willing to try VRT guided by a virtual coach or the factors that relate to this willingness.

Health care staff, when asked for their views regarding service users’ opinions of VRT, had concerns regarding patients’ willingness to accept their use as part of their mental health care package [19]. Furthermore, the literature has highlighted a lack of personalization as a barrier to engagement with digital mental health interventions [20]. It is unclear whether this indicates that automated VRTs can be sufficient when scripts are relevant to the experience of the individual. It is possible that the presence of a virtual coach may encourage more positive attitudes and a willingness to try VRTs. There is a need to understand service user and public perspectives on the use and delivery of VRTs and those guided by a virtual coach and how different factors may affect the uptake of such interventions.

### Aim

The main aim of this study was to establish a new, validated questionnaire to measure the perceptions of VRT guided by a virtual coach. Second, we aimed to explore how these perceptions are associated with familiarity with VR and mental health, using both quantitative and qualitative approaches.

## Methods

### Study Design

This study used mixed methods with a cross-sectional design. Data were collected from a web-based questionnaire using Jisc software [21].

### Participants

The participants were recruited via social media to complete the web-based questionnaire. We aimed to recruit a minimum of 200 participants in line with sample size recommendations for exploratory factor analysis (EFA) [22]. To be eligible to participate, persons were required to be a resident of the United Kingdom or Ireland and aged ≥18 years. A link to the web-based survey was included in all promotional materials.

### Measures

#### Demographics

The participants were asked to provide basic demographic information, including their age and sex, as well as whether they were identified as having a mental health condition, had ever experienced therapy, or had supported anyone with a mental health condition. Furthermore, they were asked about their experience of VR (from never to ≥10 times) and their familiarity with VR, VRT, and mental health conditions.

#### The Attitudes Towards Virtual Reality Therapy Scale

The Attitudes Towards Virtual Reality Therapy (AVRT) Scale was developed by AMA and ADGB. Items were based on themes identified in previous literature that contribute to perceptions of VRT and digital mental health interventions [14,23-29]. Items surrounding the virtual coach drew on the literature related to therapeutic alliance and focused on trust, comfort, and need [30].

We designed 54 items all assessing different aspects of attitudes toward VRT, including 9 items related to attitudes toward VRT delivered by a virtual coach. Each item used a 7-point Likert scale where participants rated their agreement from “strongly agree” to “strongly disagree.” Strong agreement or disagreement with 16 of these items triggered a free-text question for participants to provide context using free-text responses. Responses were scored from 1 (strongly disagree) to 7 (strongly agree). A higher score indicates more positive perceptions of VRT. A total of 27 items were reverse-worded and therefore reverse-coded.

Furthermore, participants were invited to respond to 3 additional free-text questions asking what they would like to know more about, how they think their level of VR experience has influenced their perceptions, and what they think the best setting for VRT would be.

### Procedure

Upon opening the questionnaire, participants were first shown the information sheet, followed by a consent statement. After consenting, participants were asked to enter a unique identification code so that their anonymized responses could be identified later. Participants were then asked to provide basic demographic information, followed by an explanatory paragraph (Multimedia Appendix 1) about VRT and the virtual coach, which was described as “a computer-generated avatar” that “guides the patients through the scenarios and offers advice and encouragement.” This was followed by items on experience with VR and mental health, the AVRT Scale, and the 3 free-text questions. After completing these questionnaires, participants were presented with a debrief statement.

### Ethics Approval

Ethics approval was granted by the Division of Psychiatry and Applied Psychology Ethics Subcommittee of the University of Nottingham (Project ID 1534).

### Statistical Analysis

Raw data were downloaded from Jisc [21] into SPSS Statistics software (version 25; IBM Corp) [31]. We removed responses from participants who did not meet the inclusion criteria, did not provide consent, or had missing data. Sample characteristics were summarized using descriptive statistics.

To validate our new questionnaire, we conducted an EFA using principal component analysis with a varimax rotation. We assessed the suitability of the data for factor analysis using Bartlett’s test of sphericity and the Kaiser-Meyer-Olkin measure of sampling adequacy (acceptable adequacy ≥0.6). All 54 items using Likert scales were included in the EFA. Items were first screened to check for multicollinearity and poor correlations with the other items. We operationalized this screening by assessing the determinant and searching for any interitem correlations of ≥0.7 or where most coefficients were nonsignificant or <0.4. Any items that failed this initial screening were removed, and the EFA was rerun. Factors were derived using eigenvalues ≥1, where the Kaiser criterion [32] was met, and in combination with the point of inflection on the scree plot, where they were not. For factors to be retained, they must comprise at least 3 items. Where items loaded on >1 factor, the item was assigned to the factor with which it made the most thematic sense.

The questionnaire included both positively and negatively worded items. Once the factors were established, we reverse-scored negatively worded items so that a higher score indicated a more favorable attitude. We then assessed the internal consistency of the final factor structure using Cronbach α, with an acceptable internal consistency of ≥0.7 [33]. Further items may be removed at this point, where the scale reliability can be substantially improved if the item is removed. The factor scores were computed using the mean and SD of the scale sum.

We conducted a series of Pearson *r* correlations to assess whether there was a relationship among the scale totals of the derived factors and lived experience of VR, VRT, and mental health problems.

### Qualitative Analysis

All responses to the free-text response questions (ie, 16 free-text boxes triggered by extreme responses to survey questions and 3 additional free-text questions) were uploaded to NVivo (version 12 for Mac; QSR International). Qualitative content analysis [34] was used to quantify and summarize the qualitative data within the broader context of the AVRT Scale. All data were coded inductively by a qualitative researcher (ADGB), where several codes could be applied to a single response. The codes were collated by questions or items. The study team met to review and revise any discrepancies or discuss any questions. Findings were then summarized according to each question or item and were presented within the factors of the AVRT Scale.

## Results

### Sample Characteristics

We collected 295 responses to the survey. Our sample reflected a range of age groups. The majority were female, had used VR at least once, and had no personal or professional experience with mental health problems. However, most participants had supported a friend or family member with poor mental health (Table 1).

**Table 1:** Sample characteristics (N=295).

Characteristics		Values
**Age group (years), n (%)**		
	18-24	89 (30.2)
	25-29	39 (13.2)
	30-39	45 (15.3)
	40-49	49 (16.6)
	50-64	64 (21.7)
	>65	9 (3.1)
**Sex, n (%)**		
	Male	83 (28.1)
	Female	209 (70.8)
	Prefer not to say	3 (1)
**Frequency of experiencing VR^a^, n (%)**		
	Never	112 (38)
	Once	48 (16.3)
	<5 times	88 (29.8)
	5-9 times	20 (6.8)
	≥10 times	27 (9.2)
**Participants identifying as having a mental health condition, n (%)**		
	Yes	102 (34.6)
	No	181 (61.4)
	Prefer not to say	12 (4.1)
**Participants with experience in therapy for a mental health condition, n (%)**		
	Yes	131 (44.4)
	No	160 (54.2)
	Prefer not to say	4 (1.4)
**Participants who have supported** **a friend or family member or colleague with a mental health condition, n (%)**		
	Yes	253 (85.8)
	No	39 (13.2)
	Prefer not to say	3 (1)
**Participants who have worked in a caring role for people with mental health conditions, n (%)**		
	Yes	108 (36.6)
	No	186 (63.1)
	Prefer not to say	1 (0.3)
Participants familiar with VR, mean (SD)		2.33 (1.02)
Participants familiar with VR therapy, mean (SD)		1.35 (0.75)
Participants familiar with mental health conditions, mean (SD)		3.65 (0.96)

^a^VR: virtual reality.

### Quantitative Analysis

#### Item Screening

The data were found to be appropriate for EFA (Kaiser-Meyer-Olkin=0.93; *χ*^2^_1431_=10,205.5, *P*<.001). The determinant suggested that there was multicollinearity, and inspection of the correlation coefficients revealed 2 pairs of items that were highly correlated (items 7 and 8=0.83; items 36 and 37=0.87); therefore, we removed 1 item from each pair of correlations (items 8 and 36). Items 13, 32, and 33 were removed, as the majority of interitem correlations were nonsignificant (*P*>.05). Furthermore, we removed items 6, 19, 25, 26, 28, 31, and 47 as either all or all but one of the correlation coefficients was <0.4. In total, we removed 12 items and then reran the EFA on the remaining 42 items.

The determinant again indicated that multicollinearity was an issue. We identified 3 pairs of correlations with coefficients >0.7 (items 10 and 11=0.76; items 29 and 49=0.76; items 35 and 37=0.71); therefore, we removed 1 item from each pair (items 10, 29, and 35) and reran the EFA on the remaining 39 items.

#### Exploratory Factor Analysis

The Kaiser criterion was met (n=295; average communalities 0.64) [32]. Therefore, the factor structure was determined based on eigenvalues >1. The rotated factor solution suggested 7 factors, which explained 63.64% of the variance. However, 3 factors were not retained because they contained <3 items. The removal of these factors resulted in the removal of items 7, 23, 24, 27, 48, and 49. The resulting 33 items were entered into a final EFA. A 4-factor solution was suggested based on the eigenvalues and the scree plot, which explained 58.61% of the variance.

Factor 1 had 13 items that assessed respondents’ support for VRT, including 6 reverse-worded items (factor 1: attitude toward VRT). Factor 2 had 9 items. These items, including 7 reverse-worded items, assessed the extent to which the respondents expected VRT to be immersive (factor 2: the expectation of presence). Factor 3 had 7 items asking respondents to compare VRT to aspects of face-to-face therapies (factor 3: preference for VRT). Factor 4 had 4 items each assessing different aspects of the cost-effectiveness of VRT (factor 4: cost-effectiveness). Refer to Multimedia Appendix 2 for the final factor structure.

#### Scale Reliability

After reverse-scoring the reverse-worded items, we computed Cronbach α values for each scale. All scales had strong internal consistency (all Cronbach α≥.82). The scale reliabilities could not be improved by removing any of the items. A higher score on each of the subscales suggested a more favorable attitude (factor 1), increased perceived presence (factor 2), a preference for VRT over traditional therapies (factor 3), and agreement that VRT is cost-effective (factor 4). The desired direction for each subscale to demonstrate support for VRT was high for factors 1, 3, and 4 and low for factor 2.

#### Relationship Between Scales and Lived Experience

There was a significant relationship between the participants’ familiarity with VR and their scores on all the factors. Familiarity with VR was positively associated with a more favorable attitude toward VRT (factor 1), higher expectations of presence (factor 2), a preference over face-to-face therapy (factor 3), and a belief that VRT is cost-effective (factor 4). We also found significant positive correlations between factors 1, 2, and 3, but not factor 4, and familiarity with the VRT. There was no significant correlation between mental health familiarity and the scores for any factors. Multimedia Appendix 2 presents the correlation coefficients and associated significance scores.

### Qualitative Results

#### Qualitative Questions

Table 2 presents the initial qualitative questions that all participants were asked.

**Table 2:** Initial qualitative questions (N=295).

Type of data	Question	Response, n (%)
Qualitative question 1	Which aspects of virtual reality therapy guided by a virtual coach would you like to know more about?	226 (76.6)
Qualitative question 2	How has your previous experience of virtual reality (minimal or extensive) influenced your perceptions of virtual reality therapy guided by a virtual coach?	236 (80)
Qualitative question 3	If you were offered virtual reality therapy guided by a virtual coach, where do you think the best place to do the therapy would be?	245 (83)

#### Which Aspects of VRT Guided by a Virtual Coach Would You Like to Know More About?

Of the 226 participants, 19 (8.4%) indicated that they did not want to know anything more. Those who provided reasons indicated that they did not want to try or did not know enough. Of them, 22 (9.7%) participants indicated that, as they did not know enough, they would like to find out more; 5 (2%) indicated that they would like to try out VRT; and 9 (3.9%) asked regarding its cost. In total, 13 (5.8%) participants were curious about the conditions that could be targeted with the VRT, specifically regarding its use for anxiety disorders, depression, and emotion regulation.

Many participants asked how it could be tailored or personalized for them (29/226, 12.8%). This meant thinking about their position within the interaction, asking about safety or how much control they would have, and whether VRT might have a negative effect and how this would be monitored. Of 226 participants, 47 (20.8%) asked about the virtual coach, wanting to know how real it would be, how much of the language would be generic or responsive to them, and how they could build a relationship with a virtual coach. Of these, many wanted more information about whether there was a real therapist involved and how involved they would be (11/47, 23%), whether they would be able to meet them in person, whether they would deliver the therapy live or preprogram the coach, or whether the virtual coach would be completely artificially intelligent. The realness of the virtual coach and the VRT (16/226, 7.1%) was also an important question posed by participants, including asking whether it would be realistic enough and comparing it to “real” or face-to-face therapy.

Most additional responses indicated that participants would like to know more about the process (67/226, 29.6%). This included practical questions regarding the frequency of use, the length of sessions, and how it would be delivered (eg, in which location). Furthermore, many participants asked about the content (26/226, 11.5%), particularly not only the scenarios that could be represented but also other aspects including the appearance, the script, and how the content could link with face-to-face therapy. Of the 226 participants, 11 (4.9%) asked about the technical aspects including the development of the coach (eg, whether an algorithm or artificial intelligence was used) and what equipment would be used to deliver the VRT.

#### How Has Your Previous Experience of VR (Minimal or Extensive) Influenced Your Perceptions of VRT Guided by a Virtual Coach?

The largest group of those answering this question indicated that they had no previous experience (79/236, 33.5%). A few without experience were positive or curious (19/236, 8.1%) whereas others (8/236, 3.1%) expressed more negative perceptions about its effectiveness as a therapeutic tool, anticipating that it would not feel real or tailored enough to the individual. The second largest group (59/236, 25%) felt that their previous experience had helped them to be more positive and linked it to their own experience of mental health and how it could be used for treatment. Although several mentioned using VR for gaming, they felt that it was effective at producing a level of presence that would be conducive to therapy and help to invoke real emotions and responses. They felt it was easy to use, could potentially lower costs, make therapy more accessible, and even with negative experiences, such as motion sickness or technical difficulties, they still had a positive perception of VRT.

However, 34 (14.4%) of the 236 participants with a more negative perception reported nausea or dizziness, whereas others perceived VR as more suited to games. This included problems with the quality of their experience, feeling that the VRT had not offered enough presence. However, those with a negative experience comprised the smallest group (8/236, 3.1%). Finally, the third largest group felt that their previous experience would not influence how they felt about VRTs guided by a virtual coach (40/236, 16.9%). For some, their previous experiences could not inform their perception of VRTs as it had been for entertainment purposes or they had too little experience to be able to make a judgment (13/236, 5.5%).

#### If You Were Offered VRT Guided by a Virtual Coach, Where Do You Think the Best Place to Do the Therapy Would Be?

The largest group of respondents who identified a single location felt that it would be best delivered within the home (84/245, 34.3%), whereas the second largest group felt that it would be best delivered in a more professional location (43/245, 17.6%). Several felt that it could be offered in both settings (38/245, 15.5%), whereas others suggested that access could first be through a clinic (12/245, 4.9%), where they could access technical or therapeutic support, or from home (4/245, 1.6%), where they would feel more comfortable. When respondents highlighted delivery from home, they described it as being safe, comfortable, and familiar. They felt that they might feel susceptible or disorientated when coming out of a VRT session and that being at home would be preferable. More professional locations, such as physician surgeries or clinics, were also described as safe and familiar, although by fewer people. Professional or clinical settings were often viewed as a better location because of the presence of support. Other reasons included the level of cleanliness offered and that there would be fewer distractions.

Those without a preference identified elements of the location that were necessary to optimize the experience, including having a space to move, feeling safe and secure (eg, in an enclosed space), having privacy and quiet, and having few distractions. They also felt it would need to consider the condition being treated (including severity) and the individual’s preferences.

### Factors With Item Responses

Table 3 presents those items where either strong agreement or disagreement elicited a qualitative response.

**Table 3:** Qualitative responses to items.

Question			Strongly agree, n (%)		Strongly disagree, n (%)
**Factor 1: attitudes toward VRT^a^ (42 and 43)**					
	Item 41: If the virtual coach encouraged me to do something between sessions, I would try to do it. (n=19)	18 (95)		1 (5)	
	Item 51: I would never be willing to try virtual reality therapy. (n=63)	3 (5)		60 (95)	
	Item 50: I would be willing to try virtual reality therapy if I had more information about it. (n=24)	21 (87)		3 (13)	
	Item 52: I would encourage the people I care about to try virtual reality therapy, if it was offered to them. (n=16)	14 (87)		2 (13)	
	Item 53: I would discourage the people I care about to try virtual reality therapy, if it was offered to them. (n=25)	1 (4)		24 (96)	
	Item 54: I cannot imagine virtual reality therapy being useful for someone with mental health problems. (n=24)	2 (8)		22 (92)	
	Item 42: I would feel comfortable interacting with the virtual coach. (n=14)	10 (71)		4 (29)	
**Factor 2: expectation of presence (47, 49, 50, 52, and 40)**					
	Item 43: I would find the characters in the virtual reality therapy unsettling. (n=6)	1 (17)		5 (83)	
	Item 45: I am skeptical about the effectiveness of virtual reality therapy. (n=13)	6 (46)		7 (54)	
	Item 44: I think that the virtual reality therapy would make me feel present enough to be effective. (n=7)	3 (43)		4 (57)	
**Factor 3: preference for VRT (54, 39, 53, and 41)**					
	Item 39: I think virtual reality therapy would be better than face-to-face therapy. (n=29)	2 (7)		27 (93)	
	Item 40: I would trust a virtual coach the same amount as a real therapist. (n=20)	4 (20)		16 (80)	
**Factor 4: cost-effectiveness (46)**					
	Item 46: I think virtual reality therapy will be worth the cost. (n=9)	6 (66)		3 (34)	
**Nonfactor answers (45, 48, 44, and 51)**					
	Item 47: I think I would be able to use the virtual reality equipment easily. (n=26)	24 (92)		2 (8)	
	Item 49: I think that virtual reality equipment could spread diseases. (n=19)	0 (0)		19 (100)	
	Item 48: If my skills with technology were poor, I would feel confident using virtual reality therapy if the health care professional accompanying me was trained to a high standard. (n=19)	17 (89)		2 (11)	

^a^VRT: virtual reality therapy.

#### Factor 1: Attitude Toward VRT

Individuals who scored highly on this factor had a positive attitude toward VRTs and VRTs delivered by a virtual coach, whereas those who scored low had a negative attitude. Within the items where strong agreement or disagreement elicited a text response (items 41, 51, 50, 52, 53, 54, and 42), those with positive attitudes highlighted the value of having a choice in mental health therapies. They emphasized the need to be willing to try different treatments to find the one that worked, reflecting on how more automated and digital options can help to increase access. Those with more negative attitudes indicated that it would be a type of therapy that they would not choose.

#### Factor 2: Expectation of Presence

Individuals who scored highly on this factor felt that VR would not be real, that is, low presence. Individuals who scored low felt that VR was immersive. Within the items where strong agreement or disagreement elicited a text response (items 43, 45, and 44), the respondents indicated several factors that affected their expectation that VR would be “real enough.” Previous experiences appeared to be linked to the expectations of presence. People who enjoyed their experiences had higher expectations of presence. Those with lower expectations felt that VRT would be too much like a game, whereas others indicated that experiencing cybersickness meant they had not felt present.

#### Factor 3: Preference for VRT

Individuals who scored highly on this factor showed a preference for VRT, whereas those who scored low showed a preference for face-to-face therapy. Within the items where strong agreement or disagreement elicited a text response (items 39 and 40), there was a strong sense that those who preferred face-to-face therapy would feel the loss of human interaction most and feel that a real person was needed to build a relationship and trust. Those who were more in favor of VRT and the virtual coach felt that it would be more convenient and potentially enable more disclosures related to their mental health.

#### Factor 4: Cost-Effectiveness

Individuals who scored high on this factor felt that VRT was cost-effective, whereas those who scored low did not. Within the item where strong agreement or disagreement elicited a text response (item 46), those who felt it was cost-effective highlighted the decreasing costs of equipment and the benefits this could bring to mental health services. For those who felt that VR was still too expensive, there was also recognition of the difficulties that services might have in adopting VR.

## Discussion

### Overview

This study aimed to develop a new instrument for assessing the public perception of VRT delivered by a virtual coach. We received 295 responses. We found that a 4-factor solution was the best fit for the AVRT Scale, with all subscales having excellent internal consistency. The 4 factors were (1) attitudes toward VRT, (2) expectation of presence, (3) preference for VRT, and (4) cost-effectiveness. We found that being more familiar with VR was correlated with more positive attitudes toward VRT (factor 1), a higher expectation of presence (factor 2), preference for VRT over face-to-face therapy (factor 3), and belief that VRT is cost-effective (factor 4). Familiarity with mental health was not associated with any factor. The qualitative data supported the quantitative findings, with many respondents stating that their previous experience with VR may have affected their perception of VRT. Respondents identified their homes and spaces that felt safe and quiet as the best locations for delivering VRT. The virtual coach was a salient concept throughout the qualitative responses, with participants wanting to better understand it and the relationships that could be facilitated.

### Principal Findings

Previous literature has indicated a correlation between VR familiarity and more positive attitudes toward VRTs [14]; however, this is the first study to demonstrate this through a public survey. Although we do not know the direction of this association, the qualitative findings suggest that as the reach of VR headsets increases, VRTs will likely be viewed more positively. The perceptions of potential patients are important in determining the efficacy of VRT, as positive expectations of any psychological therapy are associated with better treatment outcomes [35,36]. Therefore, an increase in the popularity of VR kits may indirectly improve the efficacy of VRTs.

This study is the first to explore peoples’ perceptions of VRTs guided by a virtual coach. Although most participants had no personal or professional experience of mental health therapy, many mentioned aspects relating to the virtual coach that draw parallels with “therapeutic alliance”; in psychotherapy, this denotes the importance of the relationship between the therapist and service user. In psychotherapy research, a strong therapeutic alliance is associated with better treatment outcomes [37]. The concept of therapeutic alliance has been studied more broadly in relation to VR-assisted therapies [30] and digital mental health [38]. The effects are similar but may be predictive of treatment outcomes to a lesser extent and more so predict engagement.

Understanding whether it is possible to foster a “therapeutic alliance” with a virtual coach and, if so, the nature of this relationship is something that the public is concerned with and therefore requires further investigation. Our findings provide initial insights into how therapeutic alliances may operate in VRT with a virtual coach. Respondents who showed a preference for VRTs indicated that the presence of a virtual coach would aid disclosure. This may be because of the anonymity that this form of communication offers [39]. Furthermore, it is notable that many were curious about the level of automation and formulation offered by the virtual coach. Qualitative findings from a trial of VRT guided by a virtual coach found that the presence of a member of staff helped to reinforce learning, which may suggest that certain elements of therapy require a certain level of formulation [40]. Our data suggest that the public view personalization as an important component of therapy and that VRTs can be improved by offering a certain level of formulation.

Another novelty of this study is the exploration of presence in relation to VRTs. Previous research has suggested that increasing presence can increase the effectiveness of VRT [41]. Newer VR-enabled headsets provide a greater level of presence as the quality of graphics and functionality, such as interactivity and sensitivity of sensors (eg, eye tracking), have improved significantly. Therefore, we sought to explore the importance of this sense of presence in the general population. Our findings indicate that those with a greater expectation of presence are more positive and more likely to show a preference for VRTs. Notably, our findings indicate that even those who are familiar with VR share concerns regarding the lack of presence and immersion in VRTs. This suggests that developers and researchers must continue to develop and update their intervention designs to ensure that VRTs do not become stagnant and continue to elicit a sense of presence.

Most of those who viewed VRTs positively emphasized the need for a choice to help increase access to mental health treatment. More automated VRTs have been designed considering the pressure to deliver psychological therapies in mental health services and the lack of resources to meet this need [10,11]. Our findings indicate that the public is aware of this and views VRTs guided by a virtual coach as an acceptable solution. Our respondents also indicated that VRT guided by a virtual coach would be suitable for delivery at home, further alleviating resource pressures. However, this was not the case for all participants, with a notable proportion wanting to access VRTs in a location that was safe, familiar, free of distractions, and large enough to use the VRT. The flexibility of location in delivering VRT is an important consideration for increasing access and meeting the needs of service users, especially when considering the strong links between poor mental health and housing quality [42]. The delivery location for VRT should be considered on a case-by-case basis.

A further consideration for the implementation of VRT found in our study is the importance of information. Our qualitative findings indicate that people were keen to better understand what was involved in VRT and the virtual coach. Preintervention expectations are key in managing service users’ expectations while also fostering hopefulness, which in turn improves engagement [43]. This information can also help to allay any concerns service users might have about VRT and help developers to understand their needs to improve the design and implementation of VRTs. For example, a small number of participants opposed the use of VRTs, which when expanded in our qualitative data collection, indicated certain ethical and moral concerns about its use in mental health care. All these views were valid, but a few may be rooted in misconceptions about VR or expectations about how it will be implemented. Therefore, potentially increasing the public’s awareness and understanding of VR and VRT may help to appease them and improve how it is deployed.

We found mixed findings regarding the impact of cybersickness on willingness to engage in VRT. For some participants, cybersickness would dissuade people from using VRT. However, this finding was not ubiquitous, with some saying that they were still interested in trying VRT even if they had experienced cybersickness. This contradicts previous research, suggesting cybersickness is a considerable barrier [17]. As technology progresses, cybersickness might become less important. We also included a question on hygiene, as our questionnaire was shared during the COVID-19 pandemic. However, this was excluded, suggesting that it was not a significant concern for the public.

### Limitations

The questionnaire has only been validated using the EFA. Further validation is required before we can confidently recommend its widespread use. Specifically, we must confirm the factor structure in a new sample using confirmatory factor analysis and assess its concurrent and discriminant validity. If we are able to replicate the strong psychometric properties found in this study, this questionnaire can be used to understand attitudes toward VRTs delivered by virtual coaches. The scale will also need to be adapted to contexts outside the United Kingdom, for example, by amending items and further validation.

Most of our respondents were female and had no previous experience with mental health conditions or therapy. However, men and those with more experience with mental health conditions or therapy may have different perceptions of VRTs. A recent review found that gender differences might affect the use and acceptability of VR, specifically that women are more susceptible to cybersickness and therefore may be less willing to use VR [44]. On the basis of this, it may be assumed that if we were to conduct a survey with male participants, the attitudes toward VRT guided by a virtual coach could be even more positive. We do not have any available evidence to indicate whether those who are living with or have lived with mental health conditions are likely to be more or less accepting of VRTs. Purposive sampling should be used in future studies to ensure that the views of these groups are included in future validation studies.

Furthermore, we sought text responses for strong agreement or disagreement with certain items. Notably, those with more neutral views may have offered additional insights, but we weighed this against the additional burden on respondents. This may also have led some participants to neutralize their views to avoid triggering a free-text question. However, there were no instructions in the questionnaire that responding differently removed the free-text responses. In addition, the range of scores indicated that this did not deter the participants from giving extreme answers. The qualitative data we captured were sufficient for our analysis.

Finally, the analysis of the relationship between familiarity and attitudes toward VR and VRT was correlational. We could not make any claim regarding the direction or causal nature of these associations. For example, those with more positive attitudes and a better understanding are more likely to become familiar with VR through continued use. However, our qualitative findings indicate that negative experiences with VR do not factor in a willingness to use VRT.

### Recommendations

Future research should further validate this questionnaire. Once this has been accomplished, the questionnaire could be used to investigate the factors that improve or worsen attitudes toward VRT and VRT guided by a virtual coach. For example, asking questions such as whether trying VR improves perceptions or whether increasing sales of domestic VR kits is associated with improved attitudes. It is also important to explore how these attitudes translate into behavior, that is, whether positive attitudes predict patient preferences and engagement with VRTs. The impact of the level of automation versus the formulation of the virtual coach on attitudes should also be explored, as this was a salient concept within the qualitative data. The AVRT Scale could be adapted and applied to other areas where VR is used to deliver interventions, such as behavior change interventions, or as a training tool. The questionnaire can also be used alongside treatment development, evaluation, and implementation to explore the barriers and facilitators specific to VRT and VRT guided by a virtual coach or the perceptions of certain populations to aid the translation of research into practice [45]. In the long term, any research that considers barriers to the uptake, engagement, and adoption of VRT has the potential to alleviate the demand for trained therapists in clinical settings, thus improving access to psychological therapies.

## Appendix

Multimedia Appendix 1 Description of virtual reality therapy and the virtual coach.

Multimedia Appendix 2 Factor structure and loadings.

## Abbreviations

AVRT: Attitudes Towards Virtual Reality Therapy
EFA: exploratory factor analysis
VR: virtual reality
VRT: virtual reality therapy

## Data Availability

All relevant data have been included in this publication. Researchers who would like to access the scale for further validation or adaptation may contact the corresponding author with a methodologically sound proposal.
