# Supplementary material for: Understanding Public Perceptions of Virtual Reality Psychological Therapy Using the Attitudes Towards Virtual Reality Therapy (AVRT) Scale: Mixed Methods Development Study
Source: JMIR Ment Health. 2024 Jan 12;11:e48537. doi: 10.2196/48537 (PMC10818238; doi:10.2196/48537)
Supplement: Multimedia Appendix 1 [file mental_v11i1e48537_app1.pdf]

Supplementary Material

Virtual reality (or VR) is a technology that creates a virtual environment that a user can experience audibly and visually. This is done using a headset and handheld controllers. Virtual reality is starting to be used to treat mental health conditions, such as schizophrenia and anxiety. With more people suffering from mental health problems, there is a higher demand for therapists than the supply can currently meet. Due to this, virtual reality therapy could allow people to access mental health treatments more easily. Virtual reality therapy creates scenarios for patients that would cause feelings of anxiety in real-life. This allows them to confront their fears in a safe environment. These scenarios can range from a doctor’s waiting room, to a bus, to a fear of heights. Patients work their way through the levels which become increasingly more intense. This is done with the help of a virtual coach. A computer-generated avatar guides the patients through the scenarios and offers advice and encouragement. Sessions typically last for 30 minutes each.
