# Supplementary material for: Understanding Public Perceptions of Virtual Reality Psychological Therapy Using the Attitudes Towards Virtual Reality Therapy (AVRT) Scale: Mixed Methods Development Study
Source: JMIR Ment Health. 2024 Jan 12;11:e48537. doi: 10.2196/48537 (PMC10818238; doi:10.2196/48537)
Supplement: Multimedia Appendix 2 [file mental_v11i1e48537_app2.pdf]

Table. Factor structure and loadings.

No.	Item	Factor loading				Scale, mean (SD)	Variance explained (%)	Cronbach α	VR[a] familiarity		VRT[b] familiarity		Mental health familiarity	
		1	2	3	4				Pearson *r*	*P* value	Pearson *r*	*P* value	*Pearson r*	*P* value
Factor 1: attitude toward VRT						*69.68 (11.95)*	*18.93*	*.92*	*0.35*	*<.001*	*0.21*	*<.001*	*0.11*	*.06*
41	If the virtual coach encouraged me to do something between sessions, I would try to do it.	.74												
51	I would never be willing to try virtual reality therapy.[c]	.68												
50	I would be willing to try virtual reality therapy if I had more information about it.	.67												
52	I would encourage the people I care about to try virtual reality therapy, if it was offered to them.	.67												
53	I would discourage the people I care	.65												

	about to try virtual reality therapy, if it was offered to them.[c]													
5	I would listen to the virtual coach.	.65												
34	No amount of practice would make me want to use virtual reality therapy.[c]	.63												
54	I cannot imagine virtual reality therapy being useful for someone with mental health problems.[c]	.62												
37	I am optimistic about virtual reality therapy being used in clinical settings.	.61												
42	I would feel comfortable interacting with the virtual coach.	.57												
38	I have a negative attitude toward virtual reality therapy.[c]	.55	.50[d]											
22	I think that using technology for therapy is positive.	.53												

4	I would not trust a virtual coach.[c]	.42											
**Factor 2: expectation of presence**						*38.85 (10.00)*	*16.15*	*.89*	*0.40*	*<.001*	*0.28*	*<.001*	*−0.04*	*.55*
14	I would feel like I was acting during virtual reality therapy.[c]		.69											
11	Virtual reality therapy would not be effective for me because I would be mindful that it is not real life.[c]		.67	.42[d]										
9	I do not think the virtual environment would feel real.[c]		.66											
43	I would find the characters in the virtual reality therapy unsettling.[c]		.65											
30	Potential side effects of virtual reality therapy would put me off using it.[c]		.63											
45	I am sceptical about the effectiveness of virtual reality therapy.[c]		.56	.42[d]										
44	I think that the virtual reality therapy would make me feel		.56											

	present enough to be effective.													
2	I would decline virtual reality therapy because I would prefer face-to-face therapy.[c]		.54											
12	I think that the virtual environment would feel real.		.48	.45[d]										
**Factor 3: preference for VRT**						*23.59 (6.81)*	*14.86*	*.87*	*0.25*	*<.001*	*0.14*	*.02*	*0−.06*	*.27*
39	I think virtual reality therapy would be better than face-to-face therapy.			.76										
3	I would trust a virtual coach more than a real therapist.			.73										
16	I believe that virtual reality therapy is more effective than face-to-face therapy.			.72										
17	I believe that virtual reality therapy is equally effective as face-to-face therapy.			.69										
40	I would trust a virtual coach the same amount as a real therapist.			.59										

15	I believe that virtual reality therapy is less effective than face-to-face therapy.[c]		.47[d]	.59										
1	I would choose face-to-face therapy over virtual reality therapy.[c]		.45[d]	.54										
**Factor 4: cost-effectiveness**						*20.39 (4.18)*	*8.67*	*.82*	*0.15*	*.01*	*0.10*	*.08*	*0.01*	*.89*
20	I think that using virtual reality therapy would save the NHS[e] money in the long term.				.87									
18	I think virtual reality therapy would be more cost-effective than face-to-face therapy.				.77									
21	I think that using virtual reality therapy would cost the NHS[e] more money in the long term.[c]				.76									
46	I think virtual reality therapy will be worth the cost.	.40[d]			.60									

[a]VR: virtual reality.

[b]VRT: virtual reality therapy.

[c]Reverse-worded items.
[d]Secondary loadings.
[e]NHS: National Health Service.
